# Microstructure and Mechanical Properties of In-Doped Low-Temperature SnPb Solders

**DOI:** 10.3390/ma18040886

**Published:** 2025-02-18

**Authors:** Xiaochen Xie, Pengrong Lin, Binhao Lian, Shimeng Xu, Yong Wang, Shuyuan Shi, Leqi Fu, Xiuchen Zhao

**Affiliations:** 1 School of Integrated Circuit Science and Engineering, Beihang University, Beijing 100191, China; 2 Beijing Microelectronics Technology Institute, Beijing 100076, China; 3 School of Integrated Circuits, Tsinghua University, Beijing 100084, China; 4 School of Materials Science & Engineering, Beijing Institute of Technology, Beijing 100081, China

**Keywords:** SnPb solder, In element, microstructure, melting point, mechanical property

## Abstract

In this paper, In was introduced into SnPb eutectic solder to develop a new low-temperature solder for three-dimensional packaging technology. SnPbIn solders containing 5, 10, 13, 15 and 17 wt.% In were prepared through vacuum induction melting. The effect of the addition of In on the microstructure and thermal and mechanical properties of the SnPbIn solders was investigated. The results showed that the SnPb eutectic solder consisted of Sn(ss) and Pb(ss), but when the In content was higher than 5 wt.%, the SnPbIn solder included Sn(ss) and Pb(ss) and a new InSn4 phase. Solid dissolution of the In element into Sn(ss) and Pb(ss) preferentially occurred. The melting points of the SnPbIn solders gradually decreased with the increasing addition of the In element. The melting point of the Sn-Pb-13In solder decreased to 150.5 °C, which met the requirements of 2.5D packaging. But the cast Sn-Pb-5In solder reached the best tensile strength of 48.8 MPa and elongation of 27.3%. Super-plasticity occurred in the cold-rolled SnPbIn, while the 59.9Sn35.1 Pb5In solder achieved elongation of 382.0% and 408.6%, respectively, at deformation of 70% and 90%. The super-plasticity originated from the recrystallization behavior and soft orientation.

## 1. Introduction

Electronic products are being developed towards miniaturization and multi-functionality, which requires reducing the feature size of devices and increasing the chip transistor density and integration density [1]. However, the current device feature size of 3 nm is approaching its physical limit, and the resulting quantum tunneling and short trench effect have greatly limited the progress of advanced devices. Relying solely on reducing the transistor size to meet Moore’s Law is difficult to realize due to the high research and development costs and technical difficulties [2]. Fortunately, 2.5D packaging technology provides a new method for achieving the development of advanced devices due to its low-cost, multi-functional and high-density characteristics [3]. Because the multi-stage interconnections within 2.5D packaging provide mechanical support and electrical interconnections in the vertical direction of the chip, the solder used should have different melting points, which includes high-temperature, medium-temperature and low-temperature. Generally, for the multi-stage interconnections in 2.5D packaging, weak reliability resulting from the high proportion of IMCs (intermetallic compounds) in small-sized solder joints and chip warpage are driving the rapid development of solders towards high-performance, low-temperature solders [4,5].

SnPb solders exhibit outstanding wettability, mechanical strength, fatigue resistance and reliability [6]. SnPb solders are commonly used in satellites and space stations in the aerospace field. For example, 63Sn37Pb solder is a medium-temperature solder used in packaging applications in the aerospace field [7,8]. As the diameter of the micro-bumps in the 2.5D package shrinks to less than 20 μm, the IMC content in the joint increases dramatically, especially after experiencing high-temperature reflow, which causes the performance of the solder joints to deteriorate. The traditional binary SnPb eutectic solder with a melting point of 180 °C can no longer meet the requirements of 2.5D packaging. Meanwhile, there is no commercially available low-temperature SnPb solder for the practical applications of aerospace electronics. Therefore, there is an urgent need to develop low-temperature solders based on binary SnPb eutectic solder for high-density 2.5D packaging. Alloying is an effective method for modifying the microstructure and properties of solders. Sn-57Bi eutectic alloy is a typical low-temperature solder with a melting point of 139 °C. Refs. [9,10] showed that addition of trace Ce, La and Co had no obvious effect on the melting point of the Sn-57Bi alloy, but the addition of 2 wt.% of the In or Ag elements reduced the melting point of the solder [11]. Zhang et al. [12] found that the addition of the In element can effectively lower the melting point of Sn60Pb40 solder, accompanied by an increasing solidification range. McCormack et al. [13] added 5 wt.% In to Sn-9Zn, which lowered the melting temperature from 198 to 188 °C, and the wetting behavior was similar to that of SnPb alloy. Chen et al. [14] investigated the effect of the In element on the structure and properties of Sn-Bi-based lead-free solder. The results showed that the Sn58Bi solder has a typical layered structure, and the eutectic structure consists of interphase layered white Bi and black Sn(ss)s; the number of incipient Sn-rich phases increases with the addition of the In element. When the addition of Bi is 4 wt.%, a granular Bi phase is formed. Bi and In particles are mainly distributed in the Sn matrix along the Bi phase. The influences of the In element on the microstructure and properties of Sn–Bi-based lead-free solder were investigated in Ref. [15]. With the addition of the In element, the amount of the primary Sn-rich phase increased in the Sn-Bi alloys. When the In content increased to 4%, a granular BiIn phase formed, distributed in the Sn matrix and along the Bi phase. The addition of In lowered the peak temperature and increased the reaction temperature. The tensile test results showed that the tensile strength changed slightly with an increasing addition of In, while the elongation increased remarkably first and then decreased after adding 2.5% In. The In element was confirmed to participate in the interfacial reactions forming Cu-Sn-In IMCs and affect the wettability of Sn–Bi solder on Cu substrate [16]. It can be seen that In contributes significantly to lowering the melting point of Sn-based brazing material without deteriorating the wettability of this brazing material, but the addition of In to SnPb brazing material has not yet been reported.

In this paper, In was introduced into SnPb solder to develop a new low-temperature solder with the desired performance for 2.5D packaging, and the effect of the In element on the microstructure and properties of the SnPb solder was investigated and discussed.

## 2. Materials and Methods

Solder alloys were prepared through vacuum induction melting (VIM). The raw materials, including Sn, Pb and Indium blocks (purchased from Qunwei Metal, with catalog No. 6, purity > 99.9%), were acid-washed to remove surface impurities. According to the chemical components listed in Table 1, the raw materials with different mass fractions (the total weight was controlled at about 200 g) were weighed using an electronic balance, and the raw materials were mixed using a planetary ball mill for 2 h and loaded into quartz tubes. The quartz tubes were heated in a furnace at 550~600 °C, and the furnace was evacuated with a vacuum and filled with Ar gas for protection. The eddy current effect promoted elemental mixing, and after casting them in copper molds, cast solder ingots with a uniform composition were obtained. After this, the cast solder alloys were cut into 9 mm thick pieces and were cold-rolled with deformation amounts of 70% and 90%, respectively. But the rolling distortion was less than 5% per pass.

**Figure 1:**
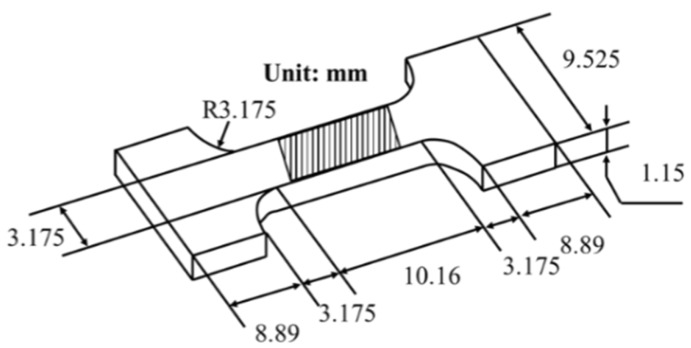
Size of tensile specimen.

The melting point and the melting range of the cast solder alloys were measured using a differential scanning calorimeter (DSC, TGA/DSC 3, Mettler Toledo, Zurich, Switzerland) at 30~300 °C. The melting point was calibrated according to the intersection between the tangent to the first data point deviating from the baseline and the tangent to the maximum slope of the DSC curve. The melting range was the difference between the peak temperature of the heat absorption peak and the melting point. The phase structure was indexed with X-ray diffraction (XRD, Ultima IV, Rigaku Corporation, Tokyo, Japan) using Cu radiation at a step of 2°/min (acquisition time per step was 0.5 s). Scanning electron microscopy (SEM, Gemini SEM 300, Carl Zeiss AG, Oberkochen, Germany) equipped with energy-dispersive X-ray spectroscopy (EDS) was used to characterize the microstructure and element contents of the solder alloys. Electron backscattered diffraction (EBSD, JSM 7001F, JEOL Ltd., Tokyo, Japan) was used to study the microstructure and texture of the solder alloys, and the data processing was conducted using TSL-OIM software (v.7.0). The static tensile properties of the cast and cold-rolled solder alloys were tested on a universal testing machine (Instron-5966, Instron, Norwood, NY, USA) at room temperature and a strain rate of 10^−3^ s^−1^. A static tensile specimen was prepared using EDM (Electrical Discharge Machining) cutting, and its shape is shown in Figure 1. Three parallel samples were tested to ensure the accuracy of the results. Figure 1 represents the location of the tensile specimen for EBSD characterization.

## 3. Results and Discussion

### 3.1. Microstructures of 63Sn37Pb Eutectic Alloy with Different Additions of In

**Figure 2:**
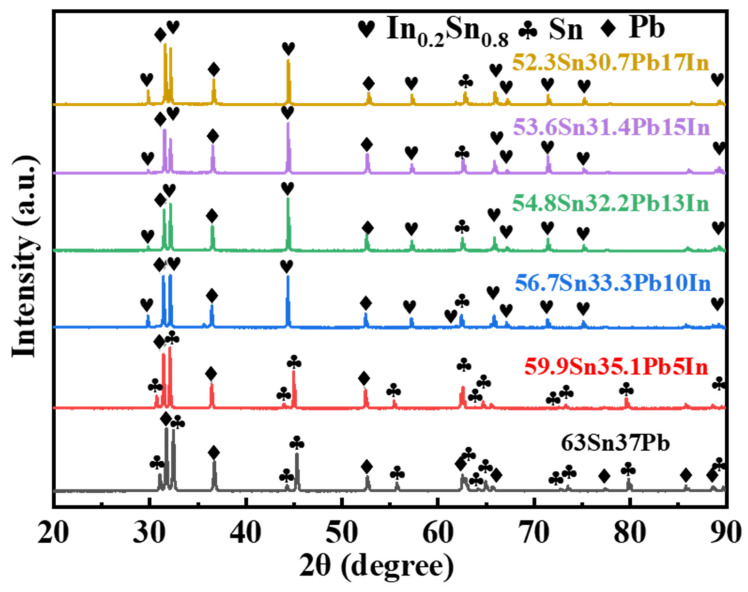
X-ray diffraction patterns of SnPbIn solders with different In additions.

Figure 2 shows the X-ray diffraction patterns of the SnPbIn solders with In additions of 0, 5, 10, 13, 15 and 17 wt.%. It can be seen that when the content of the In element was less than 10 wt.%, only the diffraction peaks of the Sn-based solid solution (Sn(ss), JCPDS card number 04-0673) and Pb-based solid solution (Pb(ss), JCPDS card number 04-001-6610) were detected. According to the In-Sn and In-Pb(ss) diagrams [17], the solid solubility of the In in the Sn(ss) and Pb(ss) is 9.7 wt.% and 57.56 wt.% below 160 °C, respectively. Therefore, the doped In element dissolved into the Sn(ss) or Pb(ss) in the Sn-Pb-5In solder. When the content of In exceeded 5 wt.%, an extra InSn4 phase was found in the SnPbIn solders. According to the In-Sn(ss) diagram [17], it is found that an InSn4 phase (IMC; Pearson symbol: hP1; space group: 191; more details can be found at https://materials.springer.com/isp/crystallographic/docs/sd_1639069 (accessed on 14 January 2025)) can form in an In-Sn system at temperatures below 119 °C, with a Sn content ranging from 76.5 to 90 at.%, and the solid solubility of In in Sn is 10 at.%, or 9.7 wt.%. When the addition of the In element exceeds 10 wt.%, In atoms become supersaturated in the Sn(ss) and form an InSn4 phase.

**Figure 3:**
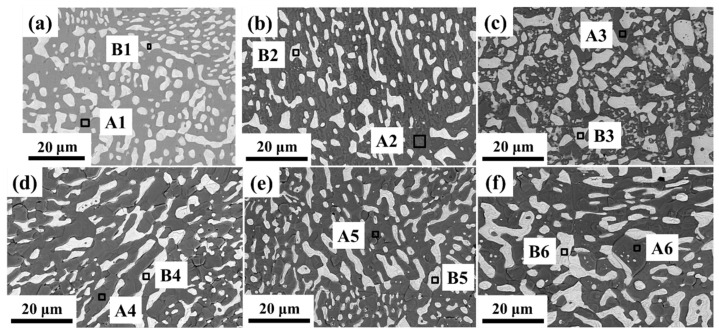
Microstructure of SnPbIn solders (The chemical composition of the regions labeled with A1-6 and B1-6 was analyzed): (**a**) 63Sn37Pb; (**b**) Sn-Pb-5In solder; (**c**) Sn-Pb-10In; (**d**) Sn-Pb-13In; (**e**) Sn-Pb-15In; and (**f**) Sn-Pb-17In.

Figure 3 and Table 2 show the microstructure and phase analysis of the SnPbIn solders with different In additions. The chemical compositions of regions A1 and B1 indicated that the 63Sn37Pb solder consisted of dark gray Sn(ss) and bright white Pb(ss). Moreover, it was observed that the Pb(ss) was diffusely distributed on the continuous Sn(ss). With an increase in the amount of In added, the In element was solidly dissolved into both the Sn and Pb(ss)s. An InSn4 phase was found in the A4-A6 region when the In content in the alloy exceeded 13%. Table 3 shows the average size and proportion of bright white Pb(ss) in Figure 3 as calculated using Image J software (v.1.53c). It could be seen that the size of the Pb(ss) in the 63Sn37Pb solder was uniform. As the content of the In element increased, the average size of the Pb(ss) gradually increased from 1.90 μm in the 63Sn37Pb solder to 6.14 μm in the Sn-Pb-17In solder, with an increase of 223.16% observed, which corresponds to the larger space between the Pb(ss)s in Figure 3. In the alloys with different contents of In, the proportion of Pb(ss) did not show an obvious change trend.

### 3.2. Thermal Properties of the 63Sn37Pb Eutectic Alloy with Different Additions of In

**Figure 4:**
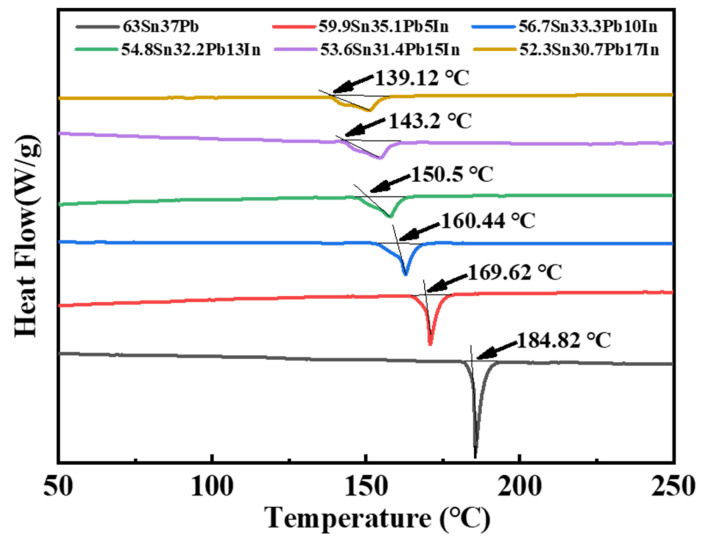
DSC curves of the SnPbIn solders with different In additions.

Figure 4 shows the DSC curves of the SnPbIn solders with In additions of 0, 5, 10, 13, 15 and 17 wt.%, respectively. The melting point of the solder was calibrated according to the intersection between the tangent to the first data point deviating from the baseline and the tangent to the maximum slope of the DSC curve. The melting range was the difference between the peak temperature of the heat absorption peak and the melting point. Table 4 lists the obtained melting points, peak temperatures and melting ranges of the SnPbIn solders. As shown in Figure 4, all of the DSC curves of the SnPbIn solders showed a single heat absorption peak. With the increase in the In content, the heat absorption peaks shifted to the left as a whole, and the heat absorption peaks gradually transformed from sharp to flat. Meanwhile, the melting point of the SnPbIn solders gradually decreased from 184.82 °C (63Sn37Pb) to 139.12 °C (Sn-Pb-17In). Compared with Sn-9Zn [13], the addition of 5% In showed a stronger effect of reducing the melting point on the Sn-PB alloys. The melting point of the In element is only 156.61 °C, which indicates that the metal bond between the In atoms is weak. The doped In element decreased the overall inter-atomic bonding and reduced the melting point of the 63Sn37Pb solder. On the other hand, the addition of the In element increased the melting range of the 63Sn37Pb solder. When the addition of In reached 17 wt.%, the melting range of the Sn-Pb-17In solder reached the maximum value of 11.81 °C, which may have been due to the presence of the InSn4 phase breaking the original Sn-Pb eutectic structure. So, as the In content increased, the proportion of the formed InSn4 phase increased, accompanied by a larger melting range.

### 3.3. Mechanical Properties of the 63Sn37Pb Eutectic Alloy with Different Additions of In

**Figure 5:**
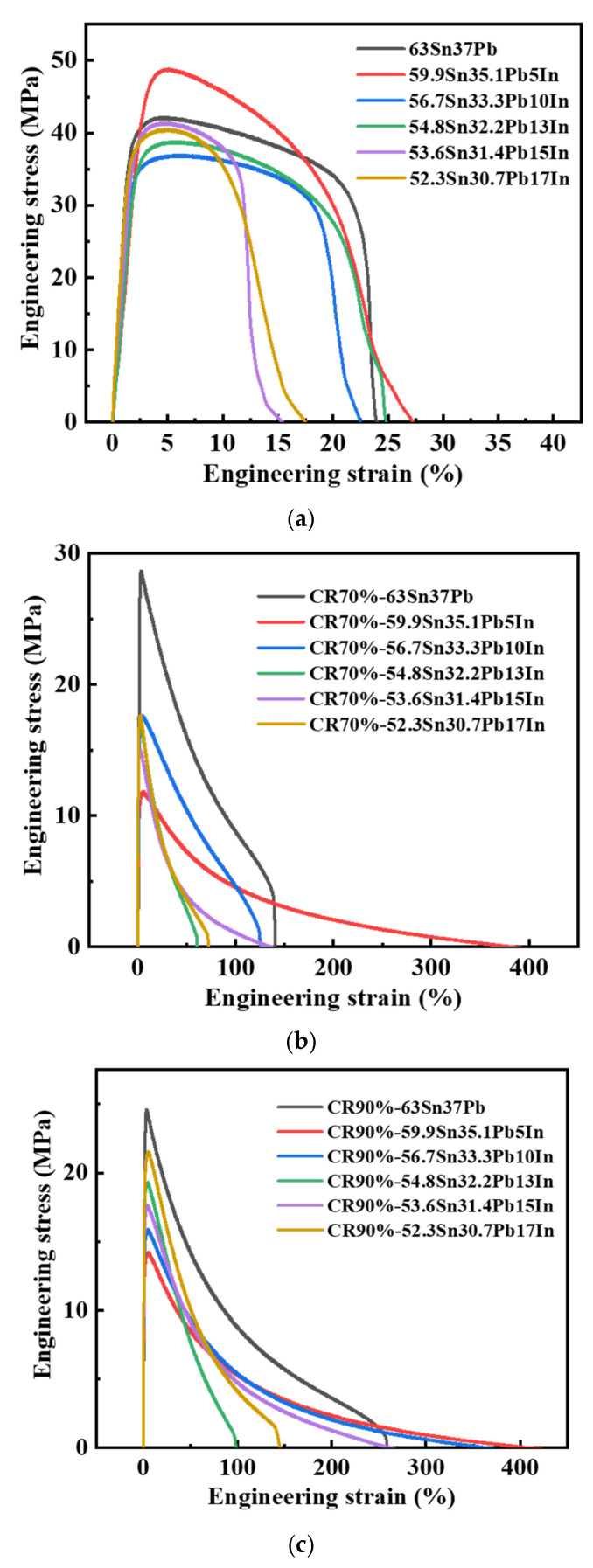
Tensile engineering stress–strain curves of SnPbIn solders. (**a**) The cast SnPbIn solders; (**b**) the rolled SnPbIn solders with different In additions under 70% cold-rolled deformation; (**c**) the rolled SnPbIn solders with different In additions under 90% cold-rolled deformation.

Figure 5a shows the tensile engineering stress–strain curves of the cast SnPbIn solders with In additions of 0, 5, 10, 13, 15 and 17 wt.%, respectively. It was observed that the ultimate tensile strength and elongation of the cast SnPbIn solders first showed an increase and then a decrease with the gradual addition of the In element. Compared with the 63Sn37Pb solder (42.2 MPa and 24.7%), the cast Sn-Pb-5In solder reached the highest ultimate tensile strength of 48.8 MPa and elongation of 27.3%, showing an increment in the ultimate tensile strength of 15.8% and increase in the elongation of 10.3%. However, when the addition of In was higher than 5 wt.%, both the ultimate tensile strength and elongation of the cast SnPbIn solder displayed a certain decline. Therefore, the Sn-Pb-5In solder not only achieved a reduced remelting point but also obtained an improvement in its mechanical properties. It represented a potential low-temperature solder. It is worth noting that the Sn-Pb-5In alloy is similar to the Sn60Pb34In6 alloy in Ref [12] in terms of its melting point (169.62 °C/166 °C) and UTS (48.2 MPa/52 MPa).

To characterize the effect of the addition of In on the mechanical properties of the SnPb alloys, the plastic deformation capacity of the SnPbIn solders was investigated. Figure 5b shows the tensile engineering stress–strain curves of the rolled SnPbIn solders with different additions of In under 70% cold-rolled deformation. It can be seen that the rolled SnPbIn solders exhibited a significantly decreased ultimate tensile strength and increased elongation compared with those of the cast SnPbIn solders. As shown in Figure 5b, rolled 63Sn37Pb had the highest ultimate tensile strength of 28.7 MPa, but the ultimate tensile strength of the rolled SnPbIn solders was only maintained in the range of 15~18 MPa. Especially, the rolled Sn-Pb-5In solder presented a maximum elongation of 382.0%, which was 171.6% higher compared with that of the 63Sn37Pb solder (140.7%). This indicated that the rolled SnPbIn solder, especially the Sn-Pb-5In solder, exhibited super-plasticity after rolling deformation of 70%.

To investigate the super-plasticity of the cold-rolled SnPbIn solder alloys further, cold rolling deformation of 90% was applied to the cast SnPnIn solders. Figure 5c shows the tensile engineering stress–strain curves of the rolled SnPbIn solders with different additions of In under 90% cold-rolled deformation. Compared with the SnPbIn solder alloys with cold rolling deformation of 70%, the SnPbIn solder alloys with cold rolling deformation of 90% displayed improved ultimate tensile strength and super-plasticity. As shown in Figure 5c, rolled 63Sn37Pb had the largest ultimate tensile strength of 24.6 MPa, but the ultimate tensile strength of the rolled SnPbIn solders was only maintained in the range of 14~22 MPa. Especially, the rolled Sn-Pb-5In solder presented a maximum elongation of 408.6%, which was 58% higher compared with that of the 63Sn37Pb solder (258.6%). Equally, the rolled Sn-Pb-10In solder also presented an improved elongation of 363.4%. This indicated that the increased cold rolling deformation improved the super-plasticity of the cast SnPbIn solders. These mechanical properties are summarized in Table 5.

**Figure 6:**
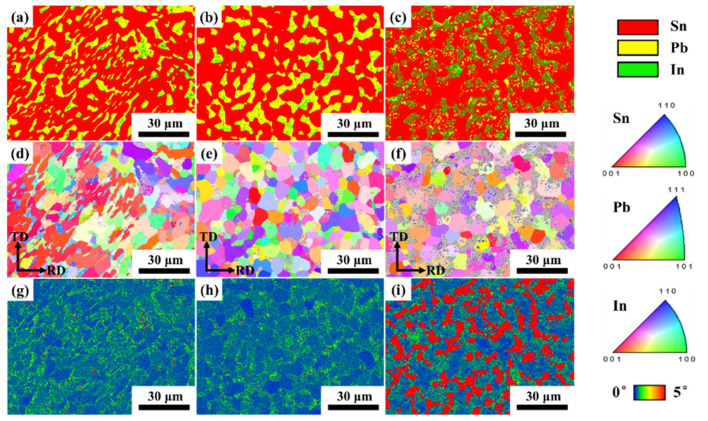
Phase maps, IPFs and KAM maps of cast and cold-rolled Sn-Pb-5In solders: (**a**–**c**) phase maps; (**d**–**f**) IPFs; (**g**–**i**) KAM maps; (**a**,**d**,**g**) cast solder; (**b**,**e**,**h**) cold rolling deformation of 70%; (**c**,**f**,**i**) cold rolling deformation of 90%.

Figure 6 shows the phase maps, inverse pole figures (IPFs) and kernel average misorientation (KAM) maps of the cast and cold-rolled Sn-Pb-5In solders. As shown in Figure 6a–c, red, yellow and green in the phase maps represent the Sn(ss), Pb(ss) and In-rich regions, respectively. In addition, the Pb(ss) mainly afforded the deformation during the cold rolling process, which is consistent with the KAM maps in Figure 6g–i. And then the Pb(ss) underwent fragmentation when the cold rolling deformation was 90%, as shown in Figure 6c. The IPF maps show that recrystallization grains appeared at the grain boundary after cold rolling. Although Figure 3 and Table 3 show that the average size of the Pb(ss) increased with an increasing In content, which was detrimental to the deformation of the Pb(ss), the cold rolling process broke up the bulk Pb(ss) and generated fine recrystallization grains at the grain boundary, which was responsible for the super-plasticity that occurred in the cold-rolled SnPbIn solders.

**Figure 7:**
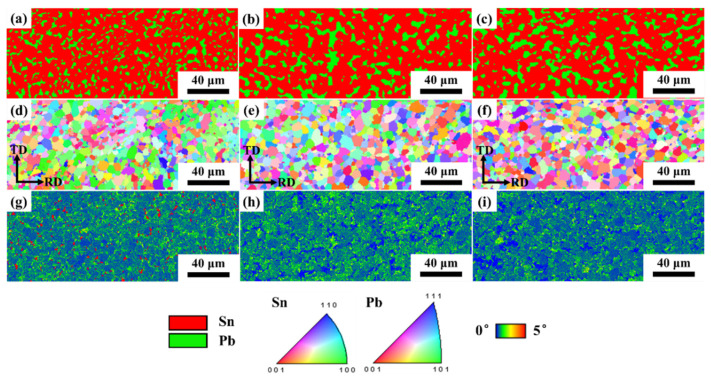
Phase maps, IPFs and KAM maps of cast and cold-rolled Sn-Pb-5In solders near tensile fracture: (**a**–**c**) phase maps; (**d**–**f**) IPFs; (**g**–**i**) KAM maps; (**a**,**d**,**g**) cast solder; (**b**,**e**,**h**) cold rolling deformation of 70%; (**c**,**f**,**i**) cold rolling deformation of 90%.

Figure 7 shows the phase maps, inverse pole figures (IPFs) and kernel average misorientation (KAM) maps of the cast and cold-rolled Sn-Pb-5In solders near tensile fracture. The detection region for EBSD is marked in Figure 1. Similarly, the red and yellow in the phase map are Sn(ss) and Pb(ss), respectively. Compared with the IPF of the SnPbIn solder (Figure 6) before tensile deformation, it can be seen that the phase distribution of the cast and rolled (deformation of 70%) solders showed no obvious change after the tensile test, but the rolled (deformation of 90%) solder underwent significant recrystallization, which was consistent with the grain size distribution shown in Figure 7d–f. And due to the occurrence of recrystallization, the KAM map of the rolled (deformation of 70% and 90%) solder, shown in Figure 7e,f, displays a low level of stress concentration.

**Figure 8:**
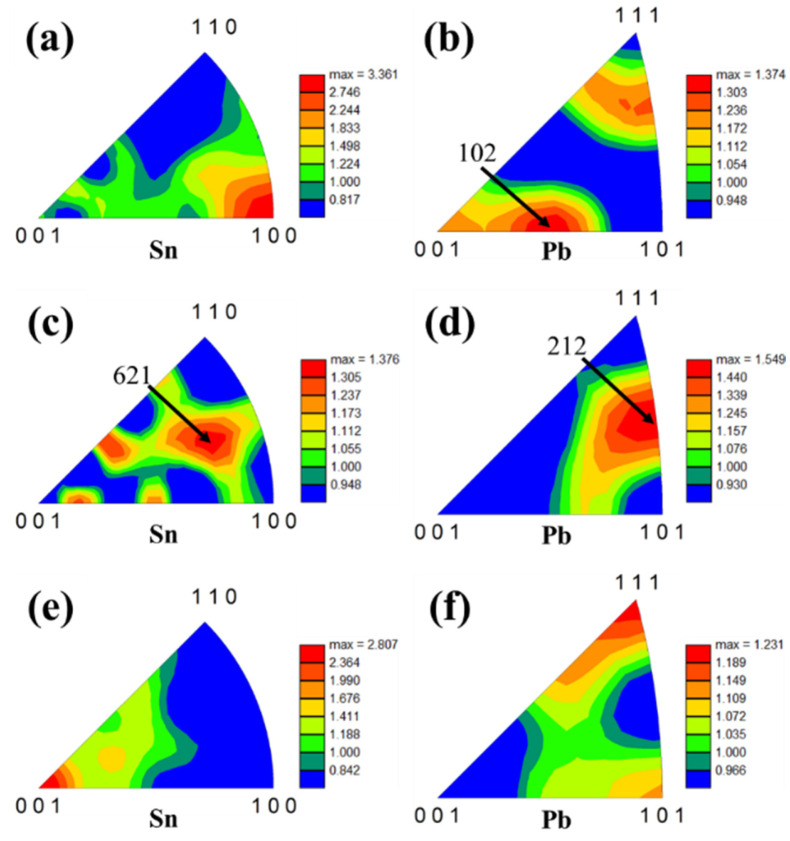
IPFs along the normal direction (ND) of the cast and rolled Sn-Pb-5In solders: (**a**,**b**) cast; (**c**,**d**) cold rolling deformation of 70%; (**e**,**f**) cold rolling deformation of 90%.

Figure 8 demonstrates the grain orientation distribution of Sn(ss) and Pb(ss) of the cast and cold-rolled Sn-Pb-5In solders after tensile deformation. It was seen that the Sn(ss) showed the (100)//ND orientation in the tensile cast solder. After cold rolling deformation, the Sn(ss) of the rolled solder showed the (621)//ND and (001)//ND orientations. Since Sn belonged to tetragonal crystals and was not involved in the tensile deformation, the orientation of Sn(ss) presented no significant change before and after cold rolling. However, the Pb(ss) of the tensile cast solder presented the (102)//ND orientation. After cold rolling deformation, the Pb(ss) of the rolled solder showed the (212)//ND and (111)//ND orientations. Since Pb is a face-centered cubic (FCC) lattice, the (111) orientation is easy to deform. In conclusion, the Sn-Pb-5In solder with cold rolling deformation of 90% exhibited recrystallization behavior and a soft orientation, which contributed to its super-plasticity. Ref [18] reported the super-plasticity phenomenon in eutectic SnPb solder at room temperature and a strain rate of 1.0 × 10^−3^ s^−1^, e.g., 430% high-pressure torsion (HPT) alloys, where a special process was employed to achieve super-plasticity by introducing dominant shear strain to cause severe plastic deformation of the alloy. The super-plasticity phenomenon in this paper was achieved through conventional cold rolling of eutectic SnPb solders, which is a process that differs considerably between the two, but the mechanism of achieving super-plasticity is generally the same.

## 4. Conclusions

(1) The 63Sn37Pb solder consisted of Sn(ss) and Pb(ss), and when the In content was higher than 5 wt.%, the SnPbIn solder included Sn(ss) and Pb(ss) and a new InSn4 phase. The In element preferentially underwent solid dissolution into the Sn(ss) and Pb(ss). This suggested that In preferentially dissolves in the solid state within both the tin and lead matrices, influencing the phase behavior of the solder.
(2) The melting points of the SnPbIn solders gradually decreased with the increasing addition of the In element. But the cast Sn-Pb-5In solder reached the best tensile strength of 48.8 MPa and elongation of 27.3%. These results highlight the potential of In as an alloying element capable of reducing the melting point and enhancing the mechanical performance of solder joints, offering promising applications in industries where strength and temperature are critical.
(3) Super-plasticity occurred in the cold-rolled SnPbIn, while the 59.9Sn35.1 Pb5In solder achieved an elongation of 382.0% and 408.6%, respectively, at a deformation of 70% and 90%. The super-plasticity originated from the recrystallization behavior and soft orientation.
(4) The addition of In has a significant effect on reducing the melting point of SnPb alloys, which is conducive to reducing the warpage of oversized 2.5D devices during the reflow process. In the future, the application of Sn-Pb-In component bumps in practical circuits will be explored, and their reliability will be studied.

## Figures and Tables

**Table 1:** Chemical components of low-temperature SnPbIn solders (wt.%).

Solders	Sn	Pb	In
63Sn37Pb	63.00	37.00	0.00
Sn-Pb-5In	59.85	35.15	5.00
Sn-Pb-10In	56.70	33.30	10.00
Sn-Pb-13In	54.81	32.19	13.00
Sn-Pb-15In	53.55	31.45	15.00
Sn-Pb-17In	52.29	30.71	17.00

**Table 2:** Phase analysis results of SnPnIn solders shown in Figure 3 (at.%).

Region	Sn	Pb	In	Possible Phases
A1	99.59	0.41	0	Sn(ss)
A2	96.73	0.06	3.21	Sn(ss)
A3	94.70	0.40	4.90	Sn(ss)
A4	86.50	0.26	13.24	InSn4
A5	85.27	0.54	14.18	InSn4
A6	84.19	0.84	14.97	InSn4
B1	3.83	96.17	0	Pb(ss)
B2	5.94	88.30	5.76	Pb(ss)
B3	4.19	89.05	6.76	Pb(ss)
B4	6.05	83.45	10.50	Pb(ss)
B5	5.64	81.66	12.71	Pb(ss)
B6	4.75	79.67	15.59	Pb(ss)

**Table 3:** Average size and proportion of Pb(ss) in the SnPnIn solders shown in Figure 3.

Solder	Average Size (μm)	Proportion of Pb(ss)
63Sn37Pb	1.9	34.2%
Sn-Pb-5In	2.7	27.8%
Sn-Pb-10In	4.3	42.1%
Sn-Pb-13In	3.2	25.3%
Sn-Pb-15In	3.7	31.2%
Sn-Pb-17In	6.1	36.7%

**Table 4:** Obtained melting point, peak temperature and melting range of the SnPbIn solders (°C).

Solder	Melting Point	Peak Temperature	Melting Range
63Sn37Pb	184.82	185.66	0.84
Sn-Pb-5In	169.62	170.92	1.30
Sn-Pb-10In	160.44	162.93	2.49
Sn-Pb-13In	150.50	157.97	7.47
Sn-Pb-15In	143.20	154.56	11.36
Sn-Pb-17In	139.12	150.93	11.81

**Table 5:** Summary of mechanical properties.

Solder	UTS (MPa)	Elongation (%)
63Sn37Pb	42.2	24.5
Sn-Pb-5In	48.2	27.5
Sn-Pb-10In	36.3	22.5
Sn-Pb-13In	38.7	25.3
Sn-Pb-15In	41.5	15.5
Sn-Pb-17In	40.6	17.4
CR70%-63Sn37Pb	28.4	141.3
CR70%-Sn-Pb-5In	11.8	387.6
CR70%-Sn-Pb-10In	17.6	124.7
CR70%-Sn-Pb-13In	16.8	59.2
CR70%-Sn-Pb-15In	15.1	140.5
CR70%-Sn-Pb-17In	17.5	72.3
CR90%-63Sn37Pb	24.3	258.4
CR90%-Sn-Pb-5In	14.2	413.1
CR90%-Sn-Pb-10In	15.9	378.6
CR90%-Sn-Pb-13In	19.0	97.8
CR90%-Sn-Pb-15In	17.6	262.4
CR90%-Sn-Pb-17In	21.8	147.6

## Data Availability

The original contributions presented in this study are included in the article. Further inquiries can be directed to the corresponding authors.
